# Intrinsic and extrinsic epigenetic age acceleration are associated with hypertensive target organ damage in older African Americans

**DOI:** 10.1186/s12920-019-0585-5

**Published:** 2019-10-22

**Authors:** Jennifer A. Smith, Jeremy Raisky, Scott M. Ratliff, Jiaxuan Liu, Sharon L. R. Kardia, Stephen T. Turner, Thomas H. Mosley, Wei Zhao

**Affiliations:** 1 Department of Epidemiology, School of Public Health, University of Michigan, Ann Arbor, MI 48109 USA; 2 Survey Research Center, Institute for Social Research, University of Michigan, Ann Arbor, MI 48104 USA; 3 Division of Nephrology and Hypertension, Mayo Clinic, Rochester, MN 55905 USA; 4 Memory Impairment and Neurodegenerative Dementia (MIND) Center, University of Mississippi Medical Center, Jackson, MS 39126 USA

**Keywords:** Epigenetic age, Age acceleration, DNA methylation, Target organ damage, Hypertension, Estimated glomerular filtration rate, Urinary albumin-creatinine ratio, Left ventricular mass, Relative wall thickness, White matter hyperintensity, Ankle-brachial index

## Abstract

**Background:**

Epigenetic age acceleration, a measure of biological aging based on DNA methylation, is associated with cardiovascular mortality. However, little is known about its relationship with hypertensive target organ damage to the heart, kidneys, brain, and peripheral arteries.

**Methods:**

We investigated associations between intrinsic (IEAA) or extrinsic (EEAA) epigenetic age acceleration, blood pressure, and six types of organ damage in a primarily hypertensive cohort of 1390 African Americans from the Genetic Epidemiology Network of Arteriopathy (GENOA) study. DNA methylation from peripheral blood leukocytes was collected at baseline (1996–2000), and measures of target organ damage were assessed in a follow-up visit (2000–2004). Linear regression with generalized estimating equations was used to test for associations between epigenetic age acceleration and target organ damage, as well as effect modification of epigenetic age by blood pressure or sex. Sequential Oligogenic Linkage Analysis Routines (SOLAR) was used to test for evidence of shared genetic and/or environmental effects between epigenetic age acceleration and organ damage pairs that were significantly associated.

**Results:**

After adjustment for sex, chronological age, and time between methylation and organ damage measures, higher IEAA was associated with higher urine albumin to creatinine ratio (UACR, *p* = 0.004), relative wall thickness (RWT, *p* = 0.022), and left ventricular mass index (LVMI, *p* = 0.007), and with lower ankle-brachial index (ABI, *p* = 0.014). EEAA was associated with higher LVMI (*p* = 0.005). Target organ damage associations for all but IEAA with LVMI remained significant after further adjustment for blood pressure and antihypertensive use (*p* < 0.05). Further adjustment for diabetes attenuated the IEAA associations with UACR and RWT, and adjustment for smoking attenuated the IEAA association with ABI. No effect modification by age or sex was observed.

**Conclusions:**

Measures of epigenetic age acceleration may help to better characterize the functional mechanisms underlying organ damage from cellular aging and/or hypertension. These measures may act as subclinical biomarkers for damage to the kidney, heart, and peripheral vasculature; however more research is needed to determine whether these relationships remain independent of lifestyle factors and comorbidities.

## Background

Nearly one third of American adults have hypertension [1], and African Americans are affected disproportionately (43% of men and 46% of women) [2]. Over time, hypertension can lead to damage in target organs, including the heart, brain, kidneys, and peripheral vasculature [3]. Recently, genome-wide association studies have begun to identify genetic risk factors for the development of specific types of hypertensive target organ damage [4–7], and large-scale epigenome-wide studies for epigenetic risk factors are underway. However, additional biomarkers are needed to better characterize and predict the type, severity, and progression of target organ damage across individuals in this era of precision medicine. A deeper understanding of etiology and enhanced risk prediction may also help to alleviate hypertension-related health disparities.

Inter-individual differences in target organ damage may be a consequence of biological, or physiological, aging due to genetic and/or environmental factors such as inflammation, oxidative stress, or other cellular mechanisms [8]. As described by Karasik et al., biological age is a measure of the functional status of an individual relative to others with the same chronological age group [9]. Biological aging can be estimated using a variety of biomarkers such as functional performance metrics (e.g., measures of frailty), blood biochemistry (e.g., inflammatory markers), telomere length, and, more recently, epigenetics. Measures of biological aging, such as telomere shortening, have been shown to be associated with subclinical measures of function or morphology in the heart [8, 10], kidneys [11], and peripheral vasculature [10]. Epigenetic mechanisms, including DNA methylation, histone acetylation, and microRNA, play a key role in the regulation of gene expression, which is known to change with age [12]. Recently, several methods have been developed that use DNA methylation, cytosine-5 methylation of CpG dinucelotides, to estimate epigenetic age acceleration, the difference between biological and chronological age [13, 14].

Epigenetic age acceleration is significantly associated with a variety of age-related conditions including all-cause mortality [15], cancer incidence and mortality [16], cardiovascular mortality [17], Alzheimer’s disease [18], Parkinson’s Disease [19], Huntington’s Disease [20], and menopause [21]. It is also associated with tissue aging [22] and cellular senescence [13, 23]. In this study, we evaluate two widely utilized measures of age acceleration, intrinsic epigenetic age acceleration (IEAA) and extrinsic epigenetic age acceleration (EEAA). IEAA captures cellular age acceleration independently of blood cell proportions, which are known to change with age. EEAA incorporates intrinsic measures as well as blood cell proportions. To date, only a few studies have investigated the association between epigenetic age acceleration and measures of target organ damage [24, 25]. It is now imperative to replicate these findings in independent populations, to examine additional measures, and to investigate whether the effects of age acceleration on target organ damage are modified by other factors.

Chronic inflammation and changes in the adaptive and innate immune response are key features of hypertension and consequent target organ damage. Peripheral blood leukocytes, which interact with peripheral tissues via excretion of cytokines and other cellular signals, are clearly implicated in target organ damage, although additional research is needed to precisely define the role of specific immune cell populations [26]. Since leukocytes provide a regulatory mechanism of inflammation due to hypertension across organ systems, peripheral blood leukocytes are an ideal cell type for exploring the relationship between hypertension and epigenetic biomarkers of age. In this study, we evaluated whether epigenetic age acceleration is associated with target organ damage to the heart, brain, kidneys, and peripheral vasculature of 1390 African Americans. For measures of target organ damage that were associated with epigenetic age acceleration, we also evaluated whether the effects were modified by systolic blood pressure (SBP), diastolic blood pressure (DBP), or sex. Finally, we used pedigree-based biometric methods to estimate heritability of the epigenetic age acceleration and target organ damage measures, and to assess whether associations between age acceleration and organ damage were due primarily to shared genetic or environmental influences.

## Methods

### Study sample

The Genetic Epidemiology Network of Arteriopathy (GENOA) study consists of hypertensive sibships that were recruited for linkage and association studies in order to identify genes that influence blood pressure and its target organ damage [27]. In the initial phase of GENOA (Phase I: 1996–2001), all members of sibships containing ≥2 individuals with essential hypertension clinically diagnosed before age 60 were invited to participate, including both hypertensive and normotensive siblings. A total of 1583 non-Hispanic whites from Rochester, MN, and 1854 African Americans from Jackson, MS, were enrolled. In the second phase of GENOA (Phase II: 2000–2004), 1239 non-Hispanic white and 1482 African American participants were successfully re-recruited to measure potential target organ damage due to hypertension. DNA methylation was measured using blood samples collected at Phase I for 1416 African Americans. After removing outliers (>4SD from the mean) and those with missing covariates, 1390 participants from 618 sibships also had measures of target organ damage collected at Phase II and/or in an ancillary study of brain MRI conducted shortly after Phase II.

### Methylation measures

Genomic DNA from 1416 participants was extracted from peripheral blood leukocytes, bisulfite converted, and then measured for DNA methylation on Illumina Infinium HumanMethylation BeadChips (*N* = 316 from 450 K and *N* = 1100 from EPIC) using stored blood samples collected at Phase I. IDAT files were imported using the *Minfi* R package [28], and the shinyMethyl R package [29] was used to exclude sex mismatches and outliers. Probes with detection *p*-value < 10^− 16^ were considered to be successfully detected [30], and samples and probes with detection rate < 10% were removed. Samples with incomplete bisulfite conversion identified using the QCinfo function in the ENmix R package [31] were removed. The proportions of each white blood cell type within the blood sample were estimated using Houseman’s method [32].

### Epigenetic age acceleration

Methylation beta values were used to estimate the DNA methylation age prior to normalization, separately for the 450 K and EPIC data, using the Horvath epigenetic age calculator (https://dnamage.genetics.ucla.edu) [13]. This calculator estimates a cumulative epigenetic aging index that predicts DNA methylation age (DNAm age). The residual from regressing DNAm age on chronological age provides a measure of epigenetic age acceleration. Horvath, et al. (2013) [13] and Hannum et al. (2013) [14] have developed independent methods that use different sets of CpG sites to estimate DNAm age, herein referred to as Horvath DNAm age and Hannum DNAm age. Intrinsic epigenetic age acceleration (IEAA) is the residual from a multivariable regression of Horvath DNAm age, estimated using 353 CpGs specified in Horvath, et al. (2013) [13], on chronological age and blood cell count estimates. This metric is independent of age-related changes in blood cell composition that are characteristic of immune system aging. It captures cell-intrinsic properties of aging with preservation across differing cell types and organs that likely indicates a fundamental aging process [33, 34].

To calculate extrinsic epigenetic age acceleration (EEAA), 71 CpGs specified in Hannum, et al. (2013) [14] are used to calculate Hannum DNAm age, which is then combined with three blood cell components (naïve cytotoxic T cells, exhausted cytotoxic T cells, and plasmablasts) to form an aggregate measure (enhanced Hannum DNAm age). EEAA is the residual from a regression of enhanced Hannum DNAm age onto chorological age. This measure captures both intrinsic epigenetic age as well as the weighted average of age-related characteristic changes in blood cell composition such as decreases in naive CD8+ T cells and increases in memory or exhausted CD8+ T cells [19]. Whereas IEAA is designed to be independent of blood cell counts, EEAA incorporates them, and is thus a measure of immune system aging.

We note that the EPIC array does not include all of the CpG sites that were originally used to construct the Horvath DNAm age (EPIC is missing 19 sites) and Hannum DNAm age (EPIC is missing 6 sites). Previous studies have demonstrated that this does not substantively compromise the performance of the DNAm age predictors [35]. Further, epigenetic age acceleration measurements such as EEAA and IEAA, which reflect the relative differences among the measured population, are especially robust to this difference in calculation.

Using the small number of samples (*n* = 102) that had methylation measured on both the 450 K and EPIC arrays, we confirmed that both epigenetic age and epigenetic age acceleration calculated from the two different array types showed relatively strong correlation (*r* = 0.88 for Horvath DNAm age, *r* = 0.95 for Hannum DNAm age, *r* = 0.70 for IEAA, *r* = 0.84 for EEAA, all *p* < 0.05). Thus, we combined data from participants with 450 K and EPIC measures of epigenetic age acceleration into a single analysis sample. For those measured on both arrays, we used the age acceleration measures calculated from the EPIC array.

### Clinical assessments and covariate definitions

Height was measured by stadiometer, and weight by electronic balance. Body mass index (BMI) was calculated as weight in kilograms divided by the square of height in meters. Resting systolic blood pressure (SBP) and diastolic blood pressure (DBP) were measured by a random zero sphygmomanometer and a cuff appropriate for arm size. The second and third of three readings, taken after the participant sat for at least 5 min, were averaged for analysis. Participants were characterized as current, former, or never smokers. Hypertension was defined as SBP ≥ 140 mmHg, DBP ≥ 90 mmHg, or self-reported physician-diagnosed hypertension and current antihypertensive medication use. Diabetes was defined as fasting serum glucose concentration ≥ 126 mg/dl or self-reported physician-diagnosed diabetes and current anti-diabetes medication use (insulin or hypoglycemic agents).

#### Kidney function

Blood was drawn after an overnight fast of at least 8 h, and urine was collected on the morning of the study visit. Serum creatinine, urine creatine, and urine albumin were assessed with enzymatic assays on a Hitachi 911 Chemistry Analyzer (Roche Diagnostics, Indianapolis, IN). Estimated glomerular filtration rate (eGFR) was calculated using serum creatinine [36]. Microalbuminuria was defined as urinary albumin-to-creatinine ratio (UACR) ≥ 30 mg/g but < 300 mg/g, and macroalbuminuria was defined as UACR ≥300 mg/g [37].

#### Ankle-brachial index (ABI)

ABI was measured from participants in the supine position following a 5-min rest [38]. Appropriately sized BP cuffs were placed on each arm and ankle, and a Doppler ultrasound instrument was used to detect pulse. The cuff was inflated to 10 mmHg above SBP and deflated at 2 mmHg/s. The first reappearance of the pulse was taken as SBP. To calculate ABI, the SBP at each site (posterior tibial and dorsalis pedis) bilaterally was divided by the higher of the two brachial SBPs (one on each side of the body). The lowest of the four ratios was designated as the ABI.

#### Echocardiography

Relative wall thickness (RWT) and left ventricular mass index (LVMI) were estimated as previously described [39]. Briefly, doppler, two-dimensional (2D), and M-mode (2D-guided) echocardiograms were performed following a standardized protocol [40]. Measurements were made at the echocardiography reading center using a computerized review station equipped with a digitizing tablet and monitor overlay used for calibration and quantification (Digisonics, Inc., Houston, Texas). Left ventricular linear dimensions were measured by M-mode or 2D echocardiography according to American Society of Echocardiography recommendations [41, 42]. Left ventricular mass (LVM) was calculated using end-diastolic dimensions by an anatomically validated formula, and RWT was calculated as twice the posterior wall thickness divided by the left ventricular internal dimension [43]. Left ventricular mass index was then calculated by dividing LVM by height in meters raised to the 2.7th power.

#### White matter hyperintensity

White matter hyperintensity (WMH) was measured at a separate study visit approximately 1 year after the Phase II examination. Brain magnetic resonance imaging (MRI) was performed using Signa 1.5 T MRI scanners (GE Medical Systems, Waukesha, WI, USA), and images were processed at Mayo Clinic [44]. Total brain and WMH volume in the corona-radiata and periventricular zone was determined from axial fluid-attenuated inversion recovery (FLAIR) images [45]. Brain scans with cortical infarctions were excluded from the analyses because of the distortion of the WMH volume estimates that would be introduced in the automated segmentation algorithm. For additional details, see Smith et al. [46].

### Statistical analysis

Target organ damage measures that were not normally distributed, UACR and WMH, were natural log transformed as ln(x) and ln(x + 1), respectively.

#### Regression modeling

Regression modeling was conducted using SAS software, Version 9.4 (Cary, NC). Generalized estimating equations (GEE) accounting for family structure were used to test the association between age acceleration (IEAA or EEAA) and blood pressure or target organ damage. Model 1 adjustment variables included age, sex, and the time between DNA methylation and target organ damage measurement. Model 2 also included SBP, DBP, and antihypertensive medication use. Model 3 further included BMI, diabetes, and smoking status. Analyses for echocardiographic measures also included microalbuminuria (300 mg/g > UACR ≥30 mg/g) and macroalbuminuria (UACR ≥300 mg/g) in Model 3 [47]. Furthermore, all models with WMH also included total intracranial volume (TIV). Age and other adjustment variables were concurrent with target organ damage measurement. If the association between epigenetic age acceleration and target organ damage lost significance in Model 3, we also evaluated which of the adjustment variables (BMI, diabetes, or smoking) was responsible for this attenuation by testing the addition of each variable separately to Model 2. For target organ damage measures that were significantly associated with age acceleration (*p* < 0.05), we examined the interaction between age acceleration and sex, SBP, or DBP using the same covariates as described previously. Since we tested six related target organ damage traits with a prior hypothesis for each of them, we were interested in significant results at both a nominal *p*-value (*p* < 0.05) as well as a Bonferroni corrected p-value (0.0083). We note that the Bonferroni approach is conservative in this setting, since the measures of target organ damage as well as the measures of epigenetic age acceleration are not independent.

#### Sensitivity analyses

Given that the full sample included individuals with epigenetic age acceleration estimated from either the EPIC or the 450 K array, we evaluated whether adding array type as a covariate and/or limiting our sample to only those individuals with EPIC data (*N* = 1074) substantively influenced our major findings. Next, since educational attainment or other markers of socioeconomic status may influence both the epigenome and the development of target organ damage, we evaluated the impact of adjusting for educational attainment (< high school, high school, college or above) in Models 1–3. Finally, since subclinical measures of damage to target organ systems may be due to hypertension as well as other age-related processes, we first conducted analyses in the full sample regardless of hypertension status. As a sensitivity analysis, we also evaluated whether the relationships between epigenetic age acceleration and target organ damage measures remained consistent in the subsample of participants with hypertension (*N* = 1127).

#### Heritability and genetic correlation

Capitalizing on the sibship structure of GENOA, Sequential Oligogenic Linkage Analysis Routines (SOLAR) v6.6.2 [48] was used to estimate the heritability of each measure of epigenetic age acceleration or target organ damage measure, adjusting for each set of covariates in Models 1–3 described above. For linear regression models that showed a significant (*p* < 0.05) association between a measure of target organ damage and epigenetic age acceleration, we investigated whether the association was due primarily to shared genetic or environmental influences. SOLAR was used to estimate the proportion of phenotypic correlation (ρP) due to shared genetic influences (genetic correlation, ρG) or environmental influences (environmental correlation, ρE) between the two traits, using the same covariates as regression Models 1–3.

## Results

Descriptive characteristics of the study participants are shown in Table 1. The average age at Phase I was 58.0 (**±**10.1) years, and the majority of the sample was female (71.1%). The average amount of time between methylation measurement (Phase I) and target organ damage measurement (Phase II) was 5.1 years, except for WMH (6.3 years). DNA methylation age was 55.0 (**±**9.9) years at Phase I. Additional file 1: Figure S1 shows scatterplots of epigenetic age and epigenetic age acceleration measures between the 450 K and EPIC arrays from 102 duplicated samples. Histograms of IEAA and EEAA are provided in Additional file 1: Figure S2.

**Table 1:** Descriptive characteristics of GENOA African Americans

Characteristic	Non-Missing *N*	Mean (SD) or %
Age at Phase I (years)	1390	58.0 (10.1)
DNAm age at Phase I (years)	1390	55.0 (9.9)
IEAA	1389	0.1 (4.8)
EEAA	1390	0.2 (5.8)
Time between Phases I and II	1390	5.1 (1.3)
Sex	1390	
Male		28.9%
Female		71.1%
Smoking	1390	
Never		60.2%
Former		27.6%
Current		12.2%
BMI (kg/m^2^)	1390	31.7 (6.7)
Systolic BP (mmHg)	1390	138.5 (20.8)
Diastolic BP (mmHg)	1390	79.5 (10.9)
Antihypertensive use	1390	
No		29.2%
Yes		70.8%
Diabetes	1390	
No		69.7%
Yes		30.3%
Albuminuria (mg/g)	1390	
Normal		81.7%
Micro^a^		14.3%
Macro^b^		4.0%
eGFR (mL/min/1.73m^2^)	1390	88.9 (21.0)
UACR (mg/g)	1390	68.4 (352.9)
RWT	1352	0.3 (0.1)
LVMI (g/m^2.7^)	1346	39.3 (10.4)
ABI	1359	1.0 (0.1)
WMH (cm^3^)^c^	758	10.6 (11.6)
TIV (cm^3^)^c^	766	1373.9 (135.8)

*Abbreviations*: *IEAA* Intrinsic epigenetic age acceleration, *EEAA* Extrinsic epigenetic age acceleration, *BMI* Body mass index, *BP* Blood pressure, *TIV* Total intracranial volume, *eGFR* Estimated glomerular filtration rate, *UACR* Urinary albumin to creatinine ratio, *LVMI* Left ventricular mass index, *RWT* Relative wall thickness, *ABI* Ankle-brachial index, *WMH* White matter hyperintensity, *TIV* Total intracranial volume

^a^Microalbuminuria is defined as 30 mg/g ≤ UACR < 300 mg/g

^b^Macroalbuminuria is defined as UACR ≥300 mg/g

^c^WMH and TIV were measured an average of 6.3 years after Phase I

All measures were taken at Phase II unless otherwise noted

Neither IEAA nor EEAA was associated with SBP or DBP in linear regression analysis, except that higher EEAA was associated with increased SBP in Model 2 (*p* = 0.015, Additional file 1: Table S1). Table 2 presents results from the association between IEAA or EEAA and each of the six target organ damage measures, separately. Higher IEAA was associated with higher UACR in Models 1 (*p* = 0.004) and 2 (*p* = 0.036); however, the association was attenuated after additional adjustment for smoking, diabetes, and BMI (Model 3, *p* = 0.246). In Model 1, a 1-year increase in IEAA was associated with 0.023 units increase in logUACR, corresponding to a 0.70 mg/g increase for a person with UACR of 30 mg/g (the threshold for microalbuminuria) and a 6.98 mg/g increase for a person with UACR of 300 mg/g (the threshold for macroalbuminuria). IEAA was not associated with eGFR.

**Table 2:** Regression results for the association between epigenetic age acceleration and target organ damage measures among GENOA African Americans

Target organ damage	Epigenetic age acceleration	Model 1		Model 2		Model 3	
		β	*P*	β	*P*	β	*P*
eGFR (*n* = 1389)	IEAA	−0.082	0.444	−0.042	0.694	−0.035	0.745
	EEAA	0.119	0.180	0.095	0.284	0.089	0.320
UACR^a^ (n = 1390)	IEAA	**0.023**	**0.004**	**0.017**	**0.036**	8.9E−3	0.246
	EEAA	0.010	0.187	7.2E-3	0.287	1.5E-3	0.826
RWT (*n* = 1352)	IEAA	**6.2E−4**	**0.022**	**5.4E-4**	**0.041**	4.5E-4	0.089
	EEAA	-3.4E-5	0.886	-4.8E-5	0.836	−1.2E-4	0.598
LVMI (*n* = 1346)	IEAA	**0.163**	**0.007**	0.110	0.054	0.062	0.260
	EEAA	**0.131**	**0.005**	**0.119**	**0.009**	**0.093**	**0.029**
ABI (*n* = 1359)	IEAA	**−2.1E-3**	**0.014**	**−1.9E-3**	**0.028**	−1.6E-3	0.057
	EEAA	−1.2E-3	0.075	−1.0E-3	0.117	−8.4E−4	0.196
WMH^a^ (*n* = 758)	IEAA	-4.2e-3	0.333	−5.4e-3	0.213	−7.8e-3	0.075
	EEAA	6.8e-3	0.068	7.0e-3	0.053	5.7e-3	0.109

*Abbreviations*: *IEAA* Intrinsic epigenetic age acceleration, *EEAA* Extrinsic epigenetic age acceleration, *eGFR* Estimated glomerular filtration rate, *UACR* Urinary albumin to creatinine ratio, *RWT* Relative wall thickness, *LVMI* Left ventricular mass index, *ABI* Ankle-brachial index, *WMH* White matter hyperintensity

Model 1: Target organ damage = epigenetic age acceleration + chronological age + sex + time between methylation and target organ damage measure

Model 2: Target organ damage = Model 1 covariates + SBP + DBP + antihypertensive medication

Model 3: Target organ damage = Model 2 covariates + smoking + diabetes + BMI

Model 3 for RWT and LVM also includes microalbuminuria and macroalbuminuria

All models for WMH also include total intracranial volume (TIV)

^a^Variables were natural log transformed prior to analysis

Bold values correspond to beta values and *p*-values, where *p* < 0.05

Higher IEAA was significantly associated with higher RWT in Models 1 and 2 (*p* = 0.022 and 0.041, respectively) and LVMI in Model 1 (*p* = 0.007). The association was only marginally significant in Model 3 for RWT (*p* = 0.089) and in Model 2 for LVMI (*p* = 0.054), and was fully attenuated in Model 3 for LVMI (*p* = 0.260). In Model 1, a 1-year increase in IEAA was associated with a 0.0006 unit increase in RWT and a 0.163 g/m^2.7^ increase in LVMI. Higher IEAA was also associated with lower ABI in Models 1 and 2 (*p* = 0.014 and 0.028, respectively), but was only marginally significant in Model 3 (*p* = 0.057). In Model 1, a 1-year increase in IEAA was associated with 0.002 units decrease in ABI. IEAA was not associated with WMH. EEAA was associated with LVMI in Models 1 (*p* = 0.005), 2 (*p* = 0.009), and 3 (*p* = 0.029), but not with other measures of organ damage. A 1-year increase in EEAA was associated with a 0.163 g/m^2.7^ increase in LVMI. Among the five associations we observed for Model 1, three (UACR and IEAA, LVMI and IEAA, LVMI and EEAA) remained significant after Bonferroni correction for multiple testing. For organ damage measures that were significantly associated with age acceleration (*p* < 0.05), we examined the interaction between age acceleration and sex, SBP, or DBP; however, none of the interactions were significant (all *p* > 0.05).

To investigate which variables were leading to the attenuation of the IEAA association with UACR, RWT, and ABI in Model 3, we examined the change in the beta coefficient for IEAA when adding each of the Model 3 adjustment variables separately to Model 2 (Additional file 1: Table S2). For UACR and RWT, adding diabetes to the model resulted in the largest percentage change of the IEAA beta coefficient (45.5 and 12.4%, respectively), resulting in attenuation of the association (*p* < 0.05). For ABI, smoking changed the beta coefficient the most (15.9%) and resulted in attenuation (*p* < 0.05).

As a sensitivity analysis, we evaluated the effect of adding array type as a covariate or excluding individuals whose epigenetic age estimates were calculated from the 450 K array (Additional file 1: Table S3). Adding array type as a covariate did not have any impact on effect estimates or *p*-values for any of the associations in Model 1. When we conducted analysis with the smaller sample measured on the EPIC array only (*N* = 1074), there are no substantive changes except that some previously significant signals are slightly attenuated (UACR and IEAA, *p* = 0.059; LVMI and IEAA, *p* = 0.062, ABI and IEAA, *p* = 0.107), which may be due to the smaller sample size. Also, the association between WMH and EEAA attained significance (*p* = 0.04) when it had previously been marginally significant (*p* = 0.068). Therefore, we conclude that combining two array types did not substantively influence the results. Adding educational attainment as a covariate also did not substantively impact the findings, except for the slight attenuation of the relationship between ABI and IEAA in Model 2 (*p* = 0.056, Additional file 1: Table S4).

We also evaluated the same linear regression models in the subset of GENOA participants with hypertension (*n* = 1127; Additional file 1: Table S5). Regression results were substantively consistent for the relationship between IEAA and UACR, LVMI, and ABI. EEAA remained associated with LVMI in Models 1 and 2 (*p* = 0.017 and *p* = 0.035), but the association was only marginally significant in Model 3 (*p* = 0.083). In contrast to the full sample, no relationship was observed between IEAA and RWT for any of the models in the subsample with hypertension. However, the association between EEAA and WMH was significant in the hypertensive sample in Models 1 and 2 (*p* = 0.040 and *p* = 0.041, respectively) and marginal in Model 3 (*p* = 0.071), whereas the associations had been only marginal in the full sample (*p* = 0.068, 0.053, and 0.109 for Models 1, 2, and 3).

All of the measures of epigenetic age acceleration and target organ damage evaluated had significant heritability (h^2^, all *p* < 0.01, Additional file 1: Table S6). In Model 1, h^2^ for IEAA was 0.480 and for EEAA was 0.607, and these estimates did not change substantially when additional covariates were added in Models 2 and 3. Of the target organ damage traits, LVMI had the highest h^2^ (0.577) and RWT had the lowest (0.276) in Model 1, and these heritabilities also remained relatively stable when additional covariates were added in Models 2 and 3. For the pairs of epigenetic age acceleration and target organ damage traits that were significantly associated in linear regression (*p* < 0.05), we also evaluated whether there was significant evidence of genetic or environmental correlation. No estimates of genetic correlation were significant (all ρG < 0.1 and *p* > 0.1), and only two of the pairs had at least marginally significant evidence of environmental correlation (ρE = 0.176 for IEAA and UACR in Model 1, *p* < 0.05; ρE = 0.229 for EEAA and LVMI in Model 1, 0.05 < *p* < 0.01; Table 3).

**Table 3:** Phenotypic, genetic, and environmental correlations between epigenetic age acceleration and target organ damage measures among GENOA African Americans

Target organ damage	Epigenetic age accleration	Model	h^2^ TOD	h^2^ EAA	ρ_P_	ρ_G_	ρ_E_
UACR^a^	IEAA	1	0.383^***^	0.484^***^	0.067^*^	−0.075	0.176^*^
		2	0.376^***^	0.510^***^	0.056^*^	0.066	0.050
RWT	IEAA	1	0.265^***^	0.483^***^	0.060^*^	0.001	0.097
		2	0.246^***^	0.510^***^	0.056^*^	0.086	0.042
LVMI	IEAA	1	0.563^***^	0.480^***^	0.064^*^	−0.032	0.168
	EEAA	1	0.566^***^	0.604^***^	0.062^*^	−0.056	0.229^†^
		2	0.506^***^	0.601^***^	0.066^*^	−0.005	0.155
		3	0.440^***^	0.604^***^	0.056^*^	−0.022	0.144
ABI	IEAA	1	0.354^***^	0.483^***^	−0.074^**^	−0.004	− 0.125
		2	0.355^***^	0.510^***^	−0.067^*^	−0.037	− 0.092

*Abbreviations*: *IEAA* Intrinsic epigenetic age acceleration, *EEAA* Extrinsic epigenetic age acceleration, *UACR* Urinary albumin to creatinine ratio, *RWT* Relative wall thickness, *LVMI* Left ventricular mass index, *ABI* Ankle-brachial index, *h*^*2*^ Heritability estimate, *ρ*_*P*_ Phenotypic correlation, *ρ*_*G*_ Genetic correlation, *ρ*_*E*_ = environmental correlation

Model 1 covariates: chronological age + sex + time between methylation and target organ damage measure

Model 2 covariates: Model 1 covariates + SBP + DBP + antihypertensive medication

Model 3 covariates: Model 2 covariates + smoking + diabetes + BMI + albuminuria

^a^Variables were natural log transformed prior to analysis

^†^*p* < 0.1, *p < 0.05, **p < 0.01, ***p < 0.001

## Discussion

Epigenetic age acceleration has shown robust associations with a variety of biological aging traits, but has not been extensively evaluated for association with organ damage associated with aging and hypertension. This study explored the association between intrinsic (IEAA) and extrinsic (EEAA) measures of age acceleration measured by DNA methylation and target organ damage in African Americans, a population disproportionately affected by hypertension. We found that IEAA was associated with measures of damage in the kidney (UACR), heart (RWT), and peripheral arterial disease (ABI), even after accounting for blood pressure and antihypertensive use. In addition, EEAA was associated with damage to the heart (LVM), and was also associated with white matter damage in the brain (WMH) for those with clinical hypertension. However, the associations between RWT and IEAA as well as ABI and IEAA were not significant after Bonferroni correction for multiple testing (Model 1); thus these results should be interpreted with caution. Neither IEAA nor EEAA was associated with eGFR, the most commonly-used biomarker of kidney function. Further, the majority of our findings were attenuated after adjusting for BMI, diabetes, and smoking, indicating that the associations between epigenetic age and target organ damage should be considered suggestive. This indicates that age acceleration may have physiological consequences, or be acting as an important subclinical biomarker for physiological processes, that influence organs vulnerable to the consequences of hypertension. However, further research is needed to determine whether these relationships remain independent of lifestyle factors and comorbidities.

For the significant associations between age acceleration and target organ damage, the effect directions were as expected. Higher IEAA, indicating a more advanced cellular aging process than expected based on chronological age, was associated with more damage to the kidney, heart, and the peripheral arteries. Adjusting for blood pressure and anti-hypertensive medications did not attenuate the significance of these associations, except for with LVMI, indicating that the relationship between IEAA and the organ damage measures was not mediated by differences in blood pressure. In fact, neither IEAA nor EEAA was strongly associated with either SBP or DBP, and relationships between IEAA and most organ damage measures remained consistent in analyses that included only those with hypertension. Thus, inter-individual differences in vulnerability to target organ damage as a result of aging and hypertension may be a consequence of cellular physiological aging processes that are relatively independent of hypertension severity as well as immune system aging (as measured by EEAA). On these points, our study is consistent with prior literature in two ways. First, that there has been little to no association observed between hypertension or blood pressure and IEAA or EEAA after controlling for BMI and lifestyle factors [17, 49]. Second, like our study, previous studies have demonstrated relationships between hypertension-related endpoints such as WMH [24] and cardiovascular mortality [50] after controlling for blood pressure level.

For target organ damage traits in the kidney (UACR) and heart (RWT), the relationship with IEAA was attenuated after adjusting for diabetes, although it remained marginally significant (*p* < 0.1) for RWT. The two primary etiologies for chronic kidney disease (CKD) are diabetic nephropathy and hypertension [51], and kidney damage due to diabetic nephropathy is often first detected clinically through increased albuminuria (higher UACR) prior to declines in eGFR [52]. Likewise, diabetes is also an independent risk factor for higher LVMI and RWT in African Americans [53], so it is possible that the relationship between these traits and IEAA was due entirely to the relationship between diabetes and IEAA. Some previous studies have found that epigenetic age acceleration was not associated with glucose and/or diabetes status after adjusting for BMI and lifestyle risk factors, and was only weakly associated with other diabetes risk factors such as triglycerides [17, 49]. A recent longitudinal study, however, observed the association between epigenetic age acceleration and fasting glucose [54]. More studies are needed to elucidate the complicated interplay among epigenetic age acceleration, diabetes, and related target organ damage to the kidney and heart.

For ABI, a 1-year change in age acceleration (IEAA) was associated with 0.002 unit decrease. Peripheral arterial disease is defined as having an ABI outside the range of 1.0–1.4 [55], with values below 1.0 indicating narrowing of arteries in certain areas of the body due to calcification [56]. The relationship between IEAA and ABI was attenuated after adjusting for smoking, which is a very strong risk factor for ABI. Even though self-reported smoking was not found to be associated with epigenetic age acceleration in previous literature [49, 57–59], many smoking-associated CpG sites were associated with methylation age acceleration [59, 60]. It has been hypothesized that biological indicators of smoking may represent susceptibility to more generalized environmental factors, including alcohol consumption and lifestyle factors, which may be partially responsible for the apparent mediating effect of smoking on the relationship between IEAA and ABI observed in this study. For both UACR and ABI, age acceleration increased variation explained by only approximately 1–2% in all models evaluated.

Few studies have evaluated the relationship between epigenetic age acceleration and target organ damage. However, two studies did find significant relationships between universal epigenetic age acceleration estimated using Horvath DNAm age acceleration and white matter abnormalities [24, 25]. In 713 African Americans aged 51 to 73, increased epigenetic age acceleration was associated with increased WMH burden, as measured by MRI, with an age acceleration increase of 1 year associated with a ~ 1 grade (0.007 on the log scale) increase of WMH [24]. In a study of 376 Mexican Americans aged 23–93 years, increased age acceleration was associated with reduced white matter integrity measured with diffusion tensor imaging [25]. These studies used measures of universal age acceleration, not IEAA and EEAA as was used in our study. However, we conducted a *post-hoc* analysis and found that universal age acceleration measures were not associated with WMH in our study (data not shown). The lack of association in our study may be due to reduced variability of the brain measures or the higher prevalence of hypertension, diabetes, or other comorbidities in our study population. However, we did observe an association between EEAA and WMH in our analysis of hypertensives only.

Although the directions of effect for IEAA and EEAA were relatively consistent in this study, they had non-overlapping significant associations with target organ damage measures. IEAA is independent of the immune blood cell counts, primarily captures cell-intrinsic methylation changes, and has been shown to be relatively independent of lifestyle factors [34]. EEAA, however, captures changes in both blood cell counts as well as cell-intrinsic methylation changes and has been shown to be more strongly correlated with lifestyle factors [34, 49]. A significant association between EEAA and LVMI may reflect the greater importance of white blood cell distributional changes that influence this trait as compared to other target organ damage measures. White blood cell distributions may be reflective of a variety of influences, including cumulative lifetime exposure to infection, lifestyle choices (diet, smoking, exercise), and social stressors (socioeconomic status, discrimination, chronic burden, neighborhood factors). In this study, we were only able to adjust for some of the potentially relevant lifestyle, social, and metabolic processes that may be associated with increased epigenetic aging.

The associations within the GENOA African American cohort are reflective of a population with a high burden of metabolic diseases, including hypertension, diabetes, and obesity. We also note that there was loss to follow-up between Phases 1 and 2 which was differential by cardiovascular risk. We found that those lost to follow-up were more likely to be older (average = 1.2 years), male, and a current smoker (all *p* < 0.05). They also had higher BMI (average = 0.58 kg/m^2^) and lower eGFR (average = 6 mL/min/1.73m^2^), and were more likely to have hypertension and diabetes (all *p* < 0.05). All of this indicates that loss to follow-up was greater in those with higher risk of cardiovascular disease, and thus our study sample may not include those with the highest risk of developing target organ damage in this population. Finally, over 2/3 of our sample consisted of women, so our results may be more relevant to this population; this is important to recognize since epigenetic aging rates in blood have been shown to be higher for men than women [17].

Previous studies have found that EEAA rates are lower for African Americans than non-Hispanic whites, but that IEAA rates are similar [17]. This is contrary to what would be expected given that African Americans experience higher rates of many age-related chronic diseases. Recently, a new DNAm age predictor has been developed that better captures the racial differences in aging that would be expected based on the disparities in health outcomes across race/ethnic groups (that is, African Americans have higher rates of epigenetic aging) [61, 62]. Future studies examining this new DNAm age predictor are warranted.

The heritability estimates of epigenetic age acceleration vary by age group, ranging from 0.39 to 0.74 in adults [13, 57, 63]. The estimated heritabilities in this study were similar (0.48 to 0.60). Although measures of epigenetic age and target organ damage had high heritability, there was very little evidence for shared genetic influences for pairs of associated epigenetic and organ damage measures. The inability to detect shared effects may be due to the relatively low correlations across pairs (all significant phenotypic correlations were less than 0.08). This may reflect the inability to adjust for all of the potentially relevant lifestyle or metabolic factors, or a true lack of pleiotropy across epigenetic age and target organ damage traits. In addition, due to the cross-sectional nature of this study, it is impossible to determine whether changes in epigenetic aging are causative or a consequence of hypertension and its subsequent target organ damage. However, there was an approximately five-year lag between the time that methylation data was collected (Phase I) and target organ damage measures (Phase II), which lends credibility to the casual nature of epigenetic aging on subsequent target organ damage. Yet we were not able to account for the duration of hypertension, which preceded epigenetic measures for over two thirds of the study population. Longitudinal studies with measures of epigenetic age at multiple timepoints may help to disentangle these temporal relationships.

An important limitation in this study was that blood pressure and target organ damage traits were measured at only one study visit. Since blood pressure is known to be highly variable and elevated during clinical visits, a better approach for accurate blood pressure measures may be to 24-h ambulatory blood pressure measures. These are available for only a subset of GENOA participants, however, so the sample size would have been prohibitory. Some of the measures of target organ damage, such as urinary albumin, are also highly variable and would be more accurate if measured on multiple days; however, UACR is a more stable biomarker than urinary albumin alone [64]. Finally, we note that antihypertensive medications have differential effects in adequately preventing or controlling target organ damage. For example, renin-angiotensin-aldosterone system-blocking agents (RAAS inhibitors) may be particularly effective in reducing damage to the kidneys and heart [64], so future studies may benefit from systematically evaluating the relationship between epigenetic age acceleration and target organ damage taking differences in antihypertensive drug classes into account.

## Conclusions

In conclusion, we have found suggestive evidence of an association with epigenetic age acceleration and target organ damage to the kidneys, heart, and peripheral arteries. Specifically, intrinsic measures of epigenetic aging were associated with urinary albumin-creatinine ratio, relative wall thickness in the heart, and ankle-brachial index. Extrinsic measures of epigenetic aging were associated with left ventricular mass index. However, most of these associations were attenuated after adjusting for blood pressure levels, BMI, diabetes, and smoking. Further research is needed to determine whether epigenetic age acceleration may have potential clinical utility in helping to quantify risk of target organ damage across organ systems.

## Supplementary information

**Additional file 1: Table S1.** Regression results for the association between epigenetic age acceleration and blood pressure in GENOA African Americans (*n* = 1390). **Table S2.** Beta coefficient for IEAA association with target organ damage measures in Model 2 and with Model 3 covariates added separately. **Table S3.** Regression results for the association between epigenetic age acceleration and target organ damage measures among GENOA African Americans, adjusting for array type or limiting analyses to those with EPIC data. **Table S4.** Regression results for the association between epigenetic age acceleration and target organ damage measures among GENOA African Americans, adjusting for educational attainment. **Table S5.** Regression results for the association between epigenetic age acceleration and target organ damage measures among GENOA African Americans with hypertension. **Table S6.** Heritability estimates of epigenetic age acceleration and target organ damage measures among GENOA African Americans. **Figure S1**. Scatterplots of DNAm age and epigenetic age acceleration from 102 duplicated samples using 450 K and EPIC array data. **Figure S2**. Distributions of epigenetic age acceleration measures.

## Data Availability

The datasets used and/or analyzed in the current study are available from the corresponding author on reasonable request.

## Abbreviations

ABI: Ankle-brachial index
BMI: Body mass index
DBP: Diastolic blood pressure
DNAm: DNA methylation
EEAA: Extrinsic epigenetic age acceleration
eGFR: Estimated glomerular filtration rate
GENOA: Genetic Epidemiology Network of Arteriopathy
IEAA: Intrinsic epigenetic age acceleration
LVMI: Left ventricular mass index
RWT: Relative wall thickness
SBP: Systolic blood pressure
SOLAR: Sequential Oligogenic Linkage Analysis Routines
UACR: Urinary albumin-creatinine ratio
WMH: White matter hyperintensity
