# Supplementary material for: Intrinsic and extrinsic epigenetic age acceleration are associated with hypertensive target organ damage in older African Americans
Source: BMC Med Genomics. 2019 Oct 22;12:141. doi: 10.1186/s12920-019-0585-5 (PMC6806502; doi:10.1186/s12920-019-0585-5)
Supplement: Supplementary file 1 — Additional file 1: Table S1. Regression results for the association between epigenetic age acceleration and blood pressure in GENOA African Americans (n = 1390). Table S2. Beta coefficient for IEAA association with target organ damage measures in Model 2 and with Model 3 covariates added separately. Table S3. Regression results for the association between epigenetic age acceleration and target organ damage measures among GENOA African Americans, adjusting for array type or limiting analyses to those with EPIC data. Table S4. Regression results for the association between epigenetic age acceleration and target organ damage measures among GENOA African Americans, adjusting for educational attainment. Table S5. Regression results for the association between epigenetic age acceleration and target organ damage measures among GENOA African Americans with hypertension. Table S6. Heritability estimates of epigenetic age acceleration and target organ damage measures among GENOA African Americans. Figure S1. Scatterplots of DNAm age and epigenetic age acceleration from 102 duplicated samples using 450 K and EPIC array data. Figure S2. Distributions of epigenetic age acceleration measures. [file 12920_2019_585_MOESM1_ESM.docx]

**Table S1.** Regression results for the association between epigenetic age acceleration and blood pressure in GENOA African Americans (n=1390)

| **Blood pressure** | **Epigenetic age acceleration** | **Model 1** | | **Model 2** | | **Model 3** | |
| --- | --- | --- | --- | --- | --- | --- | --- |
|  |  | β | *P* | β | *P* | β | *P* |
| SBP | IEAA | 0.119 | 0.321 | 0.116 | 0.176 | 0.073 | 0.401 |
|  | EEAA | 0.149 | 0.127 | 0.188 | **0.015** | 0.147 | 0.051 |
| DBP | IEAA | -6.0E-3 | 0.924 | -0.040 | 0.374 | -0.011 | 0.797 |
|  | EEAA | -0.021 | 0.678 | -0.072 | 0.073 | -0.049 | 0.203 |

Abbreviations: SBP, systolic blood pressure; DBP, diastolic blood pressure; EAA, epigenetic age acceleration; IEAA, intrinsic epigenetic age acceleration; EEAA, extrinsic epigenetic age acceleration

Model 1: Blood pressure = epigenetic age acceleration + chronological age + sex + time between methylation and blood pressure

Model 2: Blood pressure = Model 1 covariates + (SBP or DBP) + antihypertensive medication

Model 3: Blood pressure = Model 2 covariates + smoking + diabetes + BMI

P-values <0.05 are in bold.

**Table S2.** Beta coefficient for IEAA association with target organ damage measures in Model 2 and with Model 3 covariates added separately

| **Target organ damage** | **Model 2** | | **Model 2**  **+ smoking** | | | **Model 2**  **+ diabetes** | | | **Model 2**  **+ BMI** | | | **Model 2**  **+ albuminuria** | | |
| --- | --- | --- | --- | --- | --- | --- | --- | --- | --- | --- | --- | --- | --- | --- |
|  | **β** | ***P*** | **β** | ***P*** | **Δβ (%)** | **β** | ***P*** | **Δβ (%)** | **β** | ***P*** | **Δβ (%)** | **β** | ***P*** | **Δβ (%)** |
| UACR | 0.017 | **0.036** | 0.016 | **0.041** | 2.4 | 9.1E-3 | 0.233 | 45.5 | 0.016 | **0.044** | 4.3 | . | . |  |
| RWT | 5.4E-4 | **0.041** | 5.3E-4 | **0.045** | 1.4 | 4.7E-4 | **0.074** | 12.4 | 5.3E-4 | **0.044** | 2.2 | 5.2E-4 | **0.050** | 4.1 |
| ABI | -1.9E-3 | **0.028** | -1.6E-3 | 0.057 | 15.9 | -1.8E-3 | **0.035** | 4.5 | -2.0E-3 | **0.019** | -6.2 | . | . |  |

Abbreviations: IEAA, intrinsic epigenetic age acceleration; UACR, urinary albumin to creatinine ratio; RWT, relative wall thickness; ABI, ankle-brachial index

Only associations in which the beta coefficient for epigenetic age acceleration was significant (p<0.05) in Model 2 and non-significant (p>0.05) in Model 3 are shown. β and *P* represent the beta coefficient for IEAA from the specified regression model, *P* represents the corresponding p-value for the beta coefficient, and Δβ represents the percentage change in β from Model 2 to the specified model.

Model 2: Target organ damage = epigenetic age acceleration + chronological age + sex + time between methylation and target organ damage + SBP + DBP + antihypertensive medication

P-values <0.05 are in bold.

**Table S3.** Regression results for the association between epigenetic age acceleration and target organ damage measures among GENOA African Americans, adjusting for array type or limiting analyses to those with EPIC data

| **Target organ damage** | **Epigenetic age acceleration** | **Original Model 1 (N=1,416)** | | **Model 1, Adjusting for Array Type (N=1,416)** | | **Original Model 1 in EPIC only (N=1,074)** | |
| --- | --- | --- | --- | --- | --- | --- | --- |
|  |  | β | *P* | β | *P* | β | *P* |
| eGFR (n=1389) | IEAA | -0.082 | 0.444 | -0.083 | 0.440 | 1.1E-3 | 0.993 |
|  | EEAA | 0.119 | 0.180 | 0.119 | 0.180 | 0.080 | 0.430 |
| UACR* (n=1390) | IEAA | 0.023 | **0.004** | 0.023 | **0.004** | 0.017 | 0.059 |
|  | EEAA | 0.010 | 0.187 | 0.010 | 0.187 | 0.013 | 0.095 |
| RWT (n=1352) | IEAA | 6.2E-4 | **0.022** | 6.2E-4 | **0.022** | 7.1E-4 | **0.016** |
|  | EEAA | -3.4E-5 | 0.886 | -3.4E-5 | 0.885 | 1.9E-4 | 0.448 |
| LVMI (n=1346) | IEAA | 0.163 | **0.007** | 0.163 | **0.007** | 0.118 | 0.062 |
|  | EEAA | 0.131 | **0.005** | 0.131 | **0.005** | 0.171 | **0.001** |
| ABI (n=1359) | IEAA | -2.1E-3 | **0.014** | -2.1E-3 | **0.014** | -1.6E-3 | 0.107 |
|  | EEAA | -1.2E-3 | 0.075 | -1.2E-3 | 0.077 | -1.4E-3 | 0.080 |
| WMH^*^  (n=758) | IEAA | -4.2e-3 | 0.333 | -4.1E-3 | 0.340 | -3.3E-4 | 0.942 |
|  | EEAA | 6.8e-3 | 0.068 | 6.9E-3 | 0.068 | 8.0E-3 | **0.040** |

Abbreviations: IEAA, intrinsic epigenetic age acceleration; EEAA, extrinsic epigenetic age acceleration; eGFR, estimated glomerular filtration rate; UACR, urinary albumin to creatinine ratio; RWT, relative wall thickness; LVMI, left ventricular mass index; ABI, ankle-brachial index; WMH, white matter hyperintensity

Original Model 1: Target organ damage = epigenetic age acceleration + chronological age + sex + time between methylation and target organ damage measure

*Variables were natural log transformed prior to analysis.

P-values <0.05 are in bold.

| **Table S4.** Regression results for the association between epigenetic age acceleration and target organ damage measures among GENOA African Americans, adjusting for educational attainment | | | | | | | |
| --- | --- | --- | --- | --- | --- | --- | --- |
| **Target Organ Damage** | **Epigenetic Age Acceleration** | **Model 1a** | | **Model 2a** | | **Model 3a** | |
|  |  | β | *P* | β | *P* | β | *P* |
| eGFR (n=1389) | IEAA | -0.073 | 0.501 | -0.032 | 0.767 | -0.026 | 0.805 |
|  | EEAA | 0.139 | 0.122 | 0.114 | 0.204 | 0.106 | 0.241 |
| UACR* (n=1390) | IEAA | 0.022 | **0.006** | 0.016 | **0.048** | 8.1E-3 | 0.288 |
|  | EEAA | 7.8E-3 | 0.299 | 5.4E-3 | 0.427 | 6.0E-5 | 0.993 |
| RWT (n=1352) | IEAA | 6.4E-4 | **0.020** | 5.6E-4 | **0.036** | 4.7E-4 | 0.078 |
|  | EEAA | -1.7E-5 | 0.943 | -2.8E-5 | 0.904 | -9.4E-5 | 0.693 |
| LVMI (n=1346) | IEAA | 0.155 | **0.010** | 0.103 | 0.070 | 0.062 | 0.261 |
|  | EEAA | 0.119 | **0.011** | 0.110 | **0.017** | 0.092 | **0.032** |
| ABI (n=1359) | IEAA | -1.9E-3 | **0.030** | -1.6E-3 | 0.056 | -1.4E-3 | 0.087 |
|  | EEAA | -7.6E-4 | 0.271 | -6.1E-4 | 0.365 | -4.9E-4 | 0.460 |
| WMH* (n=758) | IEAA | -4.6E-3 | 0.298 | -5.8E-3 | 0.186 | -8.0E-3 | 0.071 |
|  | EEAA | 6.4E-3 | 0.094 | 6.6E-3 | 0.077 | 5.6E-3 | 0.128 |

Abbreviations: IEAA, intrinsic epigenetic age acceleration; EEAA, extrinsic epigenetic age acceleration; eGFR, estimated glomerular filtration rate; UACR, urinary albumin to creatinine ratio; RWT, relative wall thickness; LVMI, left ventricular mass index; ABI, ankle-brachial index; WMH, white matter hyperintensity

Model 1a: Target organ damage = epigenetic age acceleration + chronological age + sex + time between methylation and target organ damage measure + educational attainment

Model 2a: Target organ damage = Model 1 covariates + SBP + DBP + antihypertensive medication + educational attainment

Model 3a: Target organ damage = Model 2 covariates + smoking + diabetes + BMI + educational attainment

Model 3 for RWT and LVM also includes microalbuminuria and macroalbuminuria.

All models for WMH also include total intracranial volume (TIV).

*Variables were natural log transformed prior to analysis.

P-values <0.05 are in bold.

**Table S5.** Regression results for the association between epigenetic age acceleration and target organ damage measures among GENOA African Americans with hypertension

| **Target organ damage** | **Epigenetic age acceleration** | **Model 1** | | **Model 2** | | **Model 3** | |
| --- | --- | --- | --- | --- | --- | --- | --- |
|  |  | β | *P* | β | *P* | β | *P* |
| eGFR (n=1126) | IEAA | -0.054 | 0.647 | -0.053 | 0.647 | -0.032 | 0.785 |
|  | EEAA | 0.118 | 0.232 | 0.093 | 0.342 | 0.097 | 0.323 |
| UACR* (n=1127) | IEAA | 0.021 | **0.026** | 0.019 | **0.035** | 9.3E-3 | 0.290 |
|  | EEAA | 0.012 | 0.152 | 7.9E-3 | 0.304 | 7.4E-4 | 0.924 |
| RWT (n=1092) | IEAA | 3.7E-4 | 0.220 | 3.9E-4 | 0.176 | 3.0E-4 | 0.306 |
|  | EEAA | -1.7E-4 | 0.505 | -2.2E-4 | 0.379 | -2.9E-4 | 0.250 |
| LVMI (n=1086) | IEAA | 0.124 | 0.062 | 0.115 | 0.073 | 0.070 | 0.254 |
|  | EEAA | 0.127 | **0.017** | 0.111 | **0.035** | 0.085 | 0.083 |
| ABI (n=1097) | IEAA | -2.3E-3 | **0.020** | -2.1E-3 | **0.028** | -1.7E-3 | 0.061 |
|  | EEAA | -1.3E-3 | 0.112 | -1.0E-3 | 0.177 | -8.8E-4 | 0.246 |
| WMH* (n=592) | IEAA | -5.8E-3 | 0.226 | -5.7E-3 | 0.223 | -8.5E-3 | 0.075 |
|  | EEAA | 8.5E-3 | **0.040** | 8.2E-3 | **0.041** | 7.1E-3 | 0.071 |

Abbreviations: IEAA, intrinsic epigenetic age acceleration; EEAA, extrinsic epigenetic age acceleration; eGFR, estimated glomerular filtration rate; UACR, urinary albumin to creatinine ratio; RWT, relative wall thickness; LVMI, left ventricular mass index; ABI, ankle-brachial index; WMH, white matter hyperintensity

Model 1: Target organ damage = epigenetic age acceleration + chronological age + sex + time between methylation and target organ damage measure

Model 2: Target organ damage = Model 1 covariates + SBP + DBP + antihypertensive medication

Model 3: Target organ damage = Model 2 covariates + smoking + diabetes + BMI

Model 3 for RWT and LVM also includes microalbuminuria and macroalbuminuria.

All models for WMH also include total intracranial volume (TIV).

*Variables were natural log transformed prior to analysis.

P-values <0.05 are in bold.

**Table S6.** Heritability estimates of epigenetic age acceleration and target organ damage measures among GENOA African Americans

| **Measure** | **h^2^ in Model 1** | **h^2^ in Model 2** | **h^2^ in Model 3** |
| --- | --- | --- | --- |
| IEAA | 0.480^***^ | 0.508^***^ | 0.503^***^ |
| EEAA | 0.607^***^ | 0.601^***^ | 0.603^***^ |
| eGFR | 0.418^***^ | 0.398^***^ | 0.439^***^ |
| UACR^a^ | 0.381^***^ | 0.377^***^ | 0.331^***^ |
| RWT | 0.276^***^ | 0.259^***^ | 0.249^***^ |
| LVMI | 0.577^***^ | 0.520^***^ | 0.451^***^ |
| ABI | 0.354^***^ | 0.355^***^ | 0.317^***^ |
| WMH^a^ | 0.346^***^ | 0.323^**^ | 0.325^**^ |

Abbreviations: IEAA, intrinsic epigenetic age acceleration; EEAA, extrinsic epigenetic age acceleration; eGFR, estimated glomerular filtration rate; UACR, urinary albumin to creatinine ratio; RWT, relative wall thickness; LVMI, left ventricular mass index; ABI, ankle-brachial index; WMH, white matter hyperintensity; h^2^, heritability estimate

Model 1: Epigenetic age acceleration or target organ damage measure = chronological age + sex

Model 2: Epigenetic age acceleration or target organ damage measure = Model 1 covariates + SBP + DBP + antihypertensive medication

Model 3: Epigenetic age acceleration or target organ damage measure = Model 2 covariates + smoking + diabetes + BMI + albuminuria

^a^Variables were natural log transformed prior to analysis.

^†^p<0.1, *p<0.05, **p<0.01, ***p<0.001

**Figure S1**. Scatterplots of DNAm age and epigenetic age acceleration from 102 duplicated samples using 450K and EPIC array data


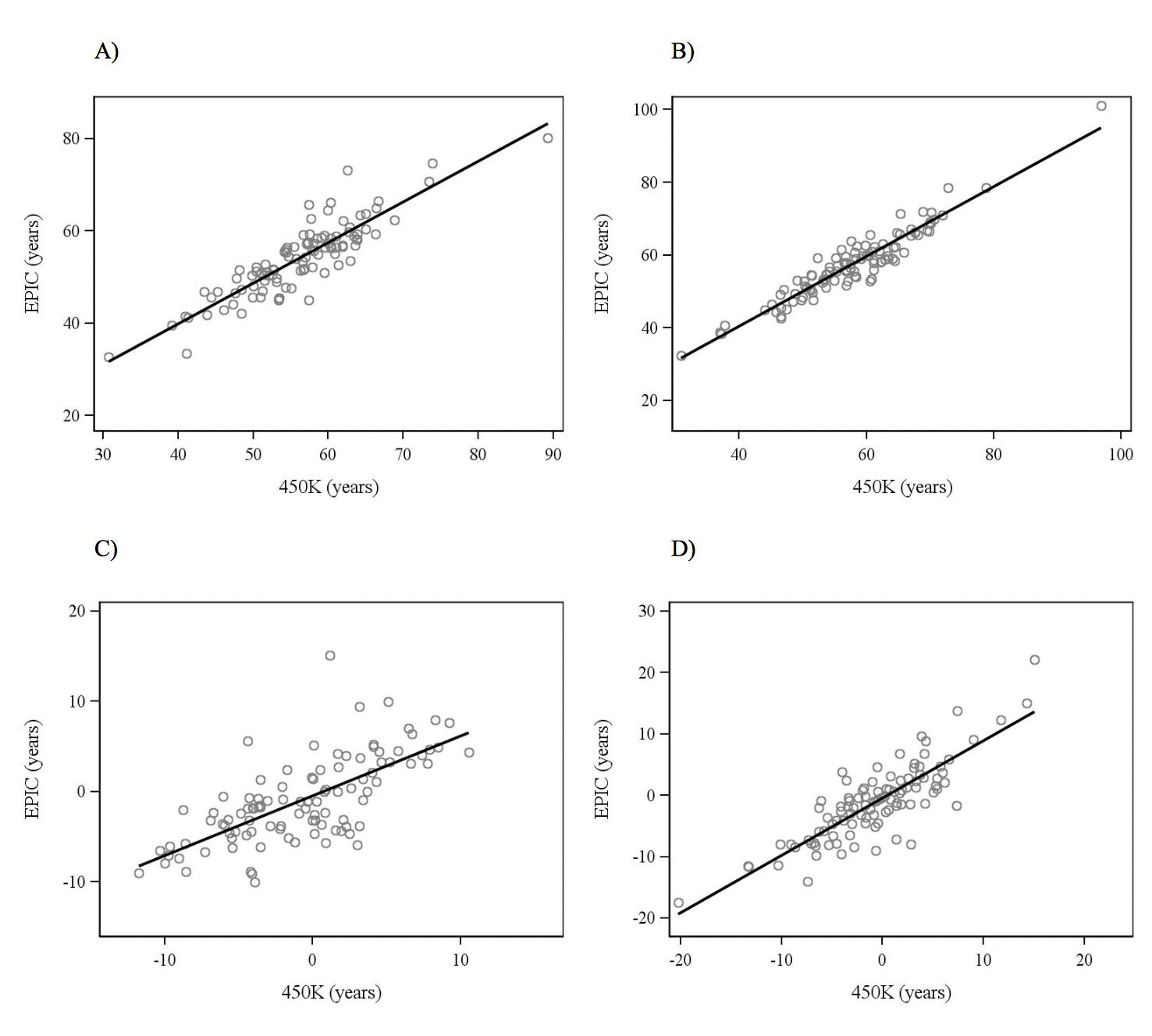


A) Horvath DNAm age (r=0.88), B) Hannum DNAm age (r=0.95), C) Intrinsic DNAm age acceleration (IEAA, r=0.70), D) Extrinsic DNAm age acceleration (EEAA, r=0.84). Linear regression lines are shown.

**Figure S2**. Distributions of epigenetic age acceleration measures

1.
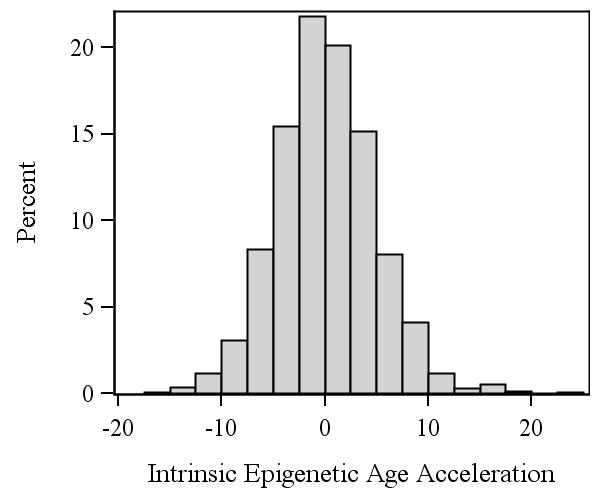

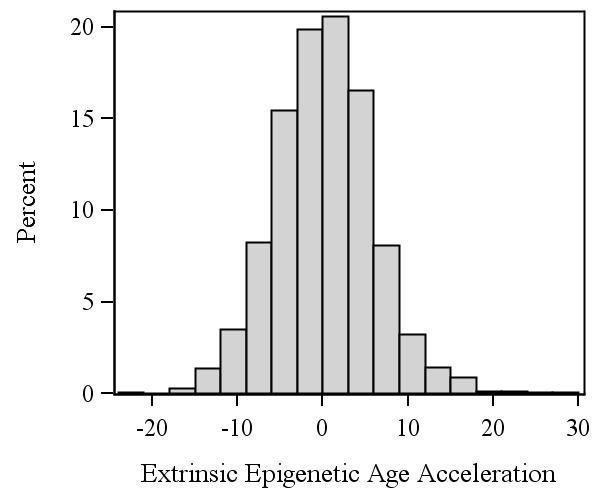
 B)

A) Intrinsic epigenetic age acceleration (IEAA); B) extrinsic epigenetic age acceleration (EEAA). Negative values indicate that the epigenetic age of the individual is younger than expected, while positive values indicate that epigenetic age is older than expected.
